# The Relationships between Cognitive Styles and Creativity: The Role of Field Dependence-Independence on Visual Creative Production

**DOI:** 10.3390/bs12070212

**Published:** 2022-06-25

**Authors:** Marco Giancola, Massimiliano Palmiero, Laura Piccardi, Simonetta D’Amico

**Affiliations:** 1 Department of Biotechnological and Applied Clinical Sciences, University of L’Aquila, 67100 L’Aquila, Italy; massimiliano.palmiero@univaq.it (M.P.); simonetta.damico@univaq.it (S.D.); 2 Department of Psychology, Sapienza University of Rome, 00185 Rome, Italy; laura.piccardi@uniroma1.it; 3 Cognitive and Motor Rehabilitation and Neuroimaging Unit, IRCCS Fondazione Santa Lucia, 00179 Rome, Italy

**Keywords:** field dependent-independent cognitive styles, creativity, creative production, Geneplore model, visual creative synthesis task

## Abstract

Previous studies explored the relationships between field dependent-independent cognitive style (FDI) and creativity, providing misleading and unclear results. The present research explored this problematic interplay through the lens of the Geneplore model, employing a product-oriented task: the Visual Creative Synthesis Task (VCST). The latter requires creating objects belonging to pre-established categories, starting from triads of visual components and consists of two steps: the preinventive phase and the inventive phase. Following the Amabile’s consensual assessment technique, three independent judges evaluated preinventive structures in terms of originality and synthesis whereas inventions were evaluated in terms of originality and appropriateness. The Embedded Figure Test (EFT) was employed in order to measure the individual’s predisposition toward the field dependence or the field independence. Sixty undergraduate college students (31 females) took part in the experiment. Results revealed that field independent individuals outperformed field dependent ones in each of the four VCST scores, showing higher levels of creativity. Results were discussed in light of the better predisposition of field independent individuals in mental imagery, mental manipulation of abstract objects, as well as in using their knowledge during complex tasks that require creativity. Future research directions were also discussed.

## 1. Introduction

Creativity has been widely recognized as the key to success in contemporary society, affecting art, science, economy, and everyday problem solving [1,2]. Given its relevance in human activities, creativity has received growing attention since the second half of the 20th century, when Guilford proposed the multifactorial Structure of Intellect Model [3], in which creative thinking encompassed convergent thinking (CT) and divergent thinking (DT). Whereas CT is stated as the ability to converge on prevailing ways of thinking in order to find a single, right, and ready-made solution to a problem that other people could also reach, DT represents a spontaneous and free-flowing form of thought and exemplifies the ability to find many new solutions to an open-ended problem. Additionally, DT is widely recognized as one of the main indicators of people’s creative potential [4], informing about the likelihood that one can act “outside the box”. Although Guilford’s work has represented a milestone in the literature of creativity, researchers have suggested alternative frameworks [5,6], including the Geneplore model [7]. The latter represents one of the most influential developments in the tradition of cognitive psychology [8], and exemplifies a twofold framework in which real-world creative production involves a cyclic motion between generative and explorative phases. The generative phase plays a crucial role in producing pre-inventive structures that are internal prototypes of inventions characterized by different degrees of creative potential and originality [9]. Whereas this phase requires several cognitive resources, including memory retrieval, mental synthesis, mental transformation, and categorical reduction [10], the explorative phase, employing cognitive and meta-cognitive processes such as attribute finding, conceptual interpretation, functional inference, and hypothesis testing, drives the examination and practical interpretation of preinventive structures to generate a creative outcome [11]. According to the product perspective [12,13], two main criteria are necessary to evaluate creative inventions: originality and appropriateness. Whereas the former encompasses the degree of novelty and uncommonness of productions, the latter refers to the relevance and usefulness, exemplifying the individual ability to produce an outcome that fits the needs and constraints of a given situation and is well situated in a given context [14]. Notably, even though different attributes for evaluating creative inventions can be found in the literature of creative production (e.g., elegance, aesthetics, and surprise), originality and appropriateness are the most accurate criteria, which reflect the full standard definition of creativity [15].

Given the multifaceted nature of creativity, the impact of cognitive factors on creative accomplishment has been long discussed in the past [16]. Cognitive style, also known as thinking style, represents a pivotal factor unquestionably related to creativity [17]. The expression cognitive style refers to how people acquire, organize, and use information [18]. Cognitive styles are usually conceptualized as bipolar, pervasive, and relatively stable over time, representing a critical dimension of the individual functioning. Although the key role of cognitive styles in human cognition and behavior has been extensively acknowledged, their universal explanatory power has not consistently been demonstrated. However, amongst all cognitive styles, the universal explanatory power of the field dependent-independent cognitive style (FDI) has been widely recognized [19]. In their seminal work, Witkin and colleagues defined FDI as “the extent to which the person perceives part of a field as discrete from the surrounding field as a whole, rather than embedded in the field” [20] (pp. 6–7). This cognitive style describes a stable and habitual tendency [21,22] characterized by two different poles: field dependence and field independence. Unlike field dependent subjects (FDs), field independent individuals (FIs) usually show less difficulty in separating information from the surrounding context [23] and are generally more focused on relevant information, inhibiting attention to irrelevant information coming from the environment [24]. Although FIs are generally defined as more flexible, open-minded, and capable of breaking down the routine than FDs, empirical evidence on the role of FDI on creativity provided unclear results [21], probably because of the involvement of specific cognitive processes (e.g., fluid intelligence, inhibition, working memory, and flexibility [25,26]), socio-cultural factors (e.g., Western vs. Eastern [27]), as well as the discrepancy between the scoring methods (e.g., empirically-based and rater-based scoring methods [28]), tasks used (e.g., divergent and convergent tasks, real-world creative production tasks [29]), and sampling bias. For a systematic review on the role of FDI in creativity, see Giancola et al. [30].

Regarding DT, some studies revealed that FIs outperformed FDs [31,32] in generating ideas, whereas others found non-significant results [33,34]. For instance, Li and colleagues [19] revealed that FIs outperformed FDs in scientific and social brainstorming tasks in terms of fluency and novelty, confirming previous research [32,35]. In addition, Lei and colleagues [17] found that field independence was related to fluency and originality but not to flexibility, whereas Niaz and colleagues [34] found no significant effect of FDI. Regarding CT, some authors stressed that FIs attained significantly higher scores than FDs in convergent measures [36,37], others found no significant effect of FDI [38]. Finally, for real-world creative production, to the knowledge of the current research, only two studies explored the impact of FDI on creative production. Specifically, Miller’s study [39] showed that FIs reported higher creativity scores than FDs in the creative collage making task. Similarly, Giancola et al. [29] found that FIs outperformed FDs in the ability to generate real-world creative objects. Notably, even though some authors found positive correlations amongst FDI and self-rated artistic abilities and artistic competencies [40,41], research on the impact of FDI on creative production remains scattered to date.

Therefore, the present research aimed to shed further light on the issue, employing the logic of the Geneplore model, which involves the combined effect of generation and exploration of ideas to generate real-world visual creative objects. Compared to Giancola et al. [29], who used a one-step procedure, priming participants with object category names while combining the visual stimuli, in the present study a two-step procedure was used. Specifically, participants were firstly instructed to construct pre-inventive forms combining the visual stimuli, and then interpret them within a specific conceptual category. This led to a better understanding of the role of FDI in the creative process, which encompasses both a visuo-spatial generative phase of undefined ideas and a conceptual/inferential phase of refined ideas. In this vein, the generative phase requires mainly divergent thinking to generate preinventive structures, whereas the explorative phase requires mainly convergent thinking to define and evaluate actual inventions [42].

Based on previous studies, the first two hypotheses were formulated as follows:

**Hypotheses** **1** **(H1).** *FIs outperform FDs in the preinventive phase because FIs are more divergent, thus more able in generating ideas than FDs [31,32];*

**Hypotheses** **2** **(H2).** *FIs outperform FDs in the inventive phase because FIs are also more convergent, thus more analytic in evaluating ideas than FDs [36,37];*

**Hypotheses** **3** **(H3).** *FIs do not outperform FDs, regardless of the phase of the creative process, because previous studies found inconsistent relationships between FDI and both divergent and convergent measures of the creative process [33,34,38].*

## 2. Materials and Methods

### 2.1. Participants and Procedure

Sixty undergraduate college students attending different courses at The University of L’Aquila (L’Aquila, Italy) participated in the study (mean age = 22.30 ± 3.31; age range = 19–32). Twenty-nine of them were males (48.3%), and thirty-one were females (51.7%). After signing the written informed consent to participate in the study, all participants were asked to complete an anamnesis questionnaire assessing biographical and educational information, general health state, and background or formal achievement in art. No participant reported psychiatric or neurological disorders, drug or alcohol addictions, and no participants declared a background or formal achievement in art. The experimental protocol was administered individually to each participant in a quiet room of the Socio-Cognitive Processes in Life Span Laboratory at The University of L’Aquila (L’Aquila, Italy). The experiment lasted approximately 45 min. The Local Ethics Committee approved this experiment in accordance with the Declaration of Helsinki.

### 2.2. Measures

#### 2.2.1. Assessment of Field Dependent Independent Cognitive Style

**Figure 1:**
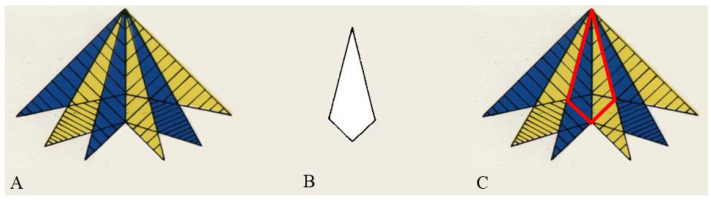
An example of an item taken from the Embedded Figure Test (EFT). (**A**) The geometric colored complex figure. (**B**) The simple black and white simple shape. (**C**) The simple shape within the complex figure.

The Embedded Figure Test—EFT [43] is usually employed to evaluate the individual’s predisposition toward the field dependent or the field independent cognitive style. It is a paper and pencil test in which participants were requested to find a simple black and white shape within a geometric colored complex figure. The test consists of 24 cards (12 cards with simple shapes and 12 cards with complex figures) 12.9 × 7.7 cm (see Figure 1).

The experimenter presented the complex-colored figure one by one for 15 s and the participant had to describe the figure in a loud voice. Then, the experimenter removed the complex figure and presented the simple one; after 10 s, he took away the simple black and white shape and presented once again the complex-colored figure. After that, participants had to find the simple black and white shape embedded in the complex figure. They were instructed to inform the experimenter as soon as they found the figure and trace its outlines using a pencil. When the participants declared to have found the simple black and white shape within the complex figure, the experimenter annotated the elapsed time (timing). If the response (tracing of the outlines) was wrong, the experimenter continued to take the time until the participant provided the correct response or until 180 s had elapsed. The total time was divided by the number of items (12) in order to compute the average time (RTs) which was used as the measure of the individual’s cognitive style. A shorter time indicated a higher predisposition towards field independence, whereas a longer time indicated a higher predisposition towards field dependence.

#### 2.2.2. Assessment of Creativity

**Figure 2:**
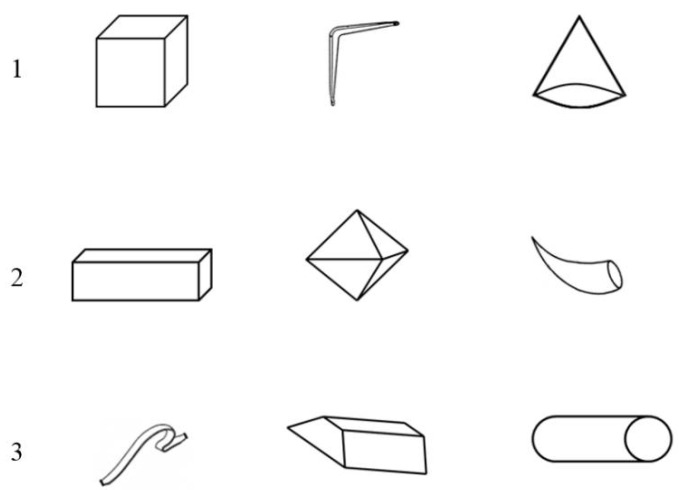
The three triads of components for the Visual Creative Synthesis Task (VCST): (**1**) cube, bracket, cone (sport goods); (**2**) parallelepiped, dy-pyramid, horn (furniture); (**3**) strip, trapezoid, cylinder (weapons).

**Figure 3:**
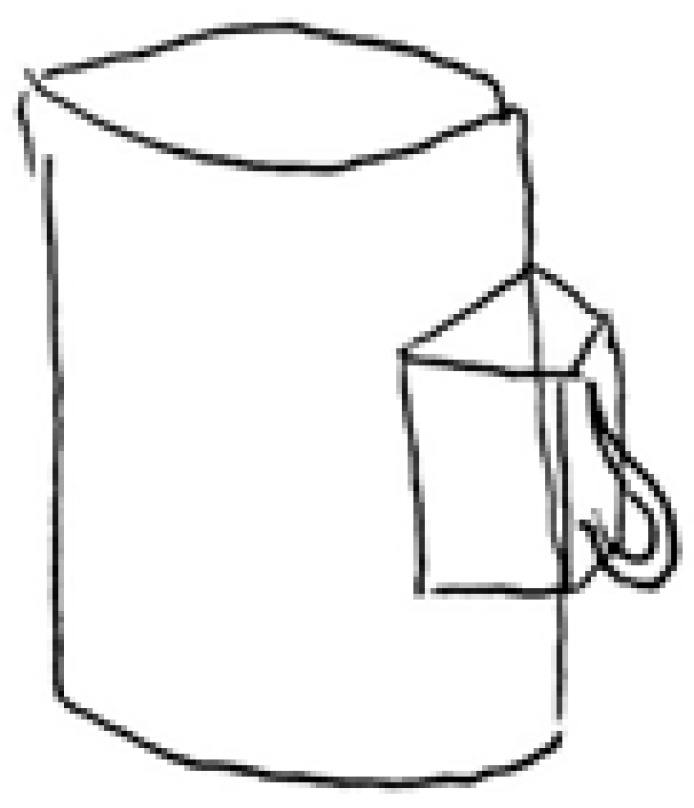
An example of creative production based on the triad n.3 made up of one stripe, one trapezoid, and one cylinder. Category: Weapons; Title: Grenade; Description: This cylinder is a grenade. The cylinder contains the explosive, the trapezoid is the trigger mechanism, and the strip controls the whole grenade and has a safety function.

The Visual Creative Synthesis Task—VCST [7] aimed to create objects belonging to pre-established categories, starting from triads of visual components. The task consists of two steps: the preinventive phase and the inventive phase. Following Palmiero and colleagues [44], three triads of components and three categories were used. Preinventive phase: participants, after a practical example, were asked to combine the components into a preinventive structure, one for each triad. They could be changed in position, rotation, and size but not in their general structure. The triads of components were presented along with their name on a paper sheet (see Figure 2). Participants had 15 s to fix and memorize the components and 2 min to think of the preinventive structure for each triad. Inventive phase: after creating the three preinventive structures, participants were presented with a category name for each of them (1 furniture, 1 weapon, and 1 sport goods) and were requested to think of their inventions. Participants had 3 min to describe the functioning of inventions (1 min for each invention), also providing their title (see Figure 3). The description of the objects was also defined in terms of length of responses (i.e., number of words). The order of the triads was randomized.

Based on Amabile’s consensual assessment technique [45], three independent judges, two females and one male (mean age = 25.33 ± 4.50), evaluated preinventive structures and inventions. The judges were three psychology students who attended training on creativity and its assessment for a total of 20 h. During the training sessions, the main models and descriptive frameworks of creativity were explained, including the SOI and the Geneplore Model. In addition, students were shown examples of creative productions already evaluated in the past by judges, and they were trained to evaluate creative productions in terms of creativity. After the training, the evaluation sessions began. Notably, three basic parameters were used: originality and appropriateness, which are the most accurate and used criteria, reflecting the full standard definition of creativity [15], as well as synthesis, which was used specifically to evaluate the participants’ ability to holistically associate the elements during the preinventive phase of the VCST. Therefore, the preinventive structures were evaluated by each judge along a 5-point Likert-type scale in terms of originality, defined as a form being new and not derived from something else (from 1 = very poor originality to 5 = very high originality) and synthesis, defined as the extent to which components were well assembled (from 1 = very poor synthesis to 5 = very high synthesis). The inter-rater correlation (intra-class correlation coefficient—absolute agreement) were significant for both originality (*α* = 0.92; *p* < 0.01) and synthesis (*α* = 0.93; *p* < 0.01). Inventions were evaluated by each judge along a 5-point Likert-type scale in terms of originality defined as a product being new and not derived from something else (from 1 = very poor originality to 5 = very high originality), and appropriateness, defined as an invention with a practical instead of a hypothetical use (from 1 = very poor appropriateness to 5 = very high appropriateness). The inter-rater correlation (intra-class correlation coefficient—absolute agreement) was significant for both originality (*α* = 0.94; *p* < 0.01) and appropriateness (*α* = 0.96; *p* < 0.01).

## 3. Results

Statistical analyses were performed using IBM SPSS Statistics version 24 for Windows (IBM Corporation, Armonk, NY, USA). Data were tested for normality and all measures were normally distributed (Kolmogorov–Smirnov Test: Z VCST—Pre—Originality = 0.75, ns; Z VCST—Pre—Synthesis = 0.73, ns; Z VCST—Inv—Originality = 0.76, ns; Z VCST—Inv—Appropriateness = 0.31, ns; Z _VCST_—_Description length_ = 0.84, ns) except for the EFT (RTs): (Kolmogorov–Smirnov Test: Z_EFT (RTs)_ = 0.02, sig). Additionally, the analysis of the distribution of the EFT using the interquartile range method (IQR) revealed that only four FDs were ‘far out’ from the mean. This suggests that in the present sample data were mostly skewed toward field dependence (e.g., high RTs) rather than field independence (e.g., low RTs). Table 1 reported descriptive statistics divided for group (field dependence vs. field independence).

Correlational analysis was computed using Spearman’s Rho (see Table 2). Results revealed that the EFT (RTs) was negatively correlated with the VCST—Preinventive phase Originality (r = −0.50, *p* < 0.01), VCST—Preinventive phase Synthesis (r = −0.48, *p* < 0.01), VCST—Inventive phase Originality (r = −0.51, *p* < 0.01) VCST—Inventive phase Appropriateness (r = −0.52, *p* < 0.01), and VCST—Description length (r = −0.30, *p* < 0.05). Notably, VCST—Description length positively correlated to VCST—Inventive phase Originality (r = 0.31, *p* < 0.05) VCST—Inventive phase Appropriateness (r = 0.29, *p* < 0.05). In addition, gender also correlated negatively to EFT (RTs) (r = −0.44, *p* < 0.01) and positively to VCST—Preinventive phase Originality (r = 0.34, *p* < 0.01), VCST—Preinventive phase Synthesis (r = 0.35, *p* < 0.01), VCST—Inventive phase Originality (r = 0.27, *p* < 0.05) and VCST—Inventive phase Appropriateness (r = 0.29, *p* < 0.05), meaning that males showed higher field dependence and creativity scores than females.

In order to obtain a more accurate picture of the relationship between EFT-RTs and the creativity scores, possible gender confounding effects were checked using the Spearman’s Rho partial correlations (see Table 3). The latter showed that controlling for gender had a little effect on the strength of the relationships between EFT-RTs and the creativity scores, and the significance level was not affected at all. Therefore, the variable ‘gender’ was not further considered when analyzing the comparison between FDs and FIs in terms of creativity.

Following Tascón and colleagues [46], since the EFT does not have a scale to divide FIs and FDs and taking into consideration that the individual’s predisposition toward field dependence and field independence is along a continuum, the median-split technique was applied. This method has also been used not only in previous studies on the interplay between FDI and creativity [36,38,47] but also in other research areas, including perception [48], spatial cognition [46], and problem solving [49]. Participants were divided by the median-split of the EFT score (average solution times). Therefore, subjects with lower scores than the median (31.23) were classified as FIs (*n* = 30), whereas participants with higher scores than the median were classified as FDs (*n* = 30).

Four Mann–Whitney U tests were performed in order to measure the differences between FDs and FIs in both preinventive and inventive phases. The significant level of the Mann–Whitney U tests was set as 5 % (α = 0.05). Results revealed that there are significant differences between FDs and FIs in terms of VCST Preinventive Originality (FDs rank = 23.83, FIs rank = 37.17, U = 250.000, *p* = 0.003), VCST Preinventive Synthesis (FDs rank = 23.47, FIs rank = 37.53, U = 239.000, *p* = 0.002), VCST Inventive Originality (FDs rank = 23.13, FIs rank = 37.87, U = 229.000, *p* = 0.001), and VCST Inventive Appropriateness (FDs rank = 23.35, FIs rank = 37.65, U = 235.000, *p* = 0.001).

## 4. Discussion

Previous research on the relationships between FDI and creativity revealed unclear results, demonstrating a lack of consensus amongst researchers [21]: mixed findings have been found taking into account creative thinking in terms of convergent [37,38] and divergent productions [32,34], whereas little work has been done on creative production [39]. Given this scenario, the current research was aimed at investigating the extent to which the individual’s predisposition towards field dependence and field independence affects creativity. To this aim, we measured FDI using the Embedded Figure Test, whereas creativity was evaluated by the VCST according to the logic of the Geneplore model. The VCST is a product-oriented task, which relies on preinventive and inventive phases. For the first time in this research field, we adopted such a twofold model, assuming that creative production requires both ideas generation and ideas evaluation, underpinned by divergent and convergent mental processes, respectively [4]. Specifically, the VCST relies on a generative process in which individuals produce preinventive structures characterized by different degrees of creative potential and on an exploratory process in which such structures are evaluated by considering their potential fruition analytically [9].

Regarding the preinventive phase, results revealed that FI negatively correlated to originality and synthesis. This result was also confirmed by the ANOVA, showing that FIs provided significantly higher scores in originality and synthesis than FDs, meaning that the faster were participants in identifying the simple shape embedded in the complex figure (field independence), the more original and well-assembled were their preinventive structures. Thus, the H1 was confirmed. Given the nature of the task used, it is not surprising that mental imagery plays a key role during the preinventive phase of VCST: indeed, the task requires mentally transforming, combining, and synthesizing visual components in order to generate preinventive structures, that is, mental prototypes of inventions. The pivotal role of mental imagery in creative tasks such as the VCST has been widely recognized in the past [10,50,51,52]. More specifically, spatial imagery and mental manipulation of spatial forms seem to be crucial in tasks involving objects’ construction [53], including creative inventions. Indeed, the ability to mentally manipulate shapes was found positively related with the originality score of preinventive structures [54] and the ability to generate shapes that were well-assembled and synthesized [10,55]. The role of spatial manipulation in creative tasks is also consistent with those studies using the think-aloud method in order to reveal mental processes actively involved in creativity. For instance, Palmiero and Piccardi’s study [56] revealed that spatial thoughts—containing spatial information of size and rotation—generated during the preinventive phase positively predicted the originality of productions during the invention phase. Although we did not detect mental imagery directly, the assumptions reported above could represent a relevant point to explain our results. Indeed, FIs seem to be more skilled than FDs in spatial abilities implying mental imagery. Specifically, FIs showed higher performance than FDs in tasks required to process different objects’ features such as shape and orientation [57] and in tasks tapping visual-spatial information [58]. For instance, Boccia and colleagues [59], in a sample of 50 young adults, found that FIs outperformed FDs in mental rotation test and Li and colleagues [60] revealed similar results in 2D and 3D map mental rotation, underlining that regardless of map dimensionality, as the degree of the image rotation increased, the accuracy of the FIs’ performance increased. Although the more flexible mental imagery and the better predisposition to use visual stimuli of FIs could represent a pivotal factor in this phase of the Geneplore cycle, undoubtedly, different mechanisms could affect it, and further investigations are needed.

Regarding the inventive phase, results revealed that FDI negatively correlated with originality and appropriateness. This result was also confirmed by the ANOVA, showing that FIs provided significantly higher scores in originality and appropriateness than FDs, meaning that the faster participants were in identifying the simple shape embedded in the complex figure (field independence), the more creative (original and appropriate) were their inventions. Results confirmed H2 and align with previous studies on real-world creative production [29,39] as well as research on both divergent thinking [17,19,32] and convergent thinking [36,37]. Two main explanations can explain the better performance of FIs in the VCST than FDs. First, the assumption of the pivotal role of mental imagery in the preinventive phase can be also extended to the inventive phase. For instance, Roskos-ewoldsen and colleagues [54], in a sample composed of 41 young and 41 older adults, found a positive relationship between the Paper Folding Test and originality score of productions in the Creative Invention Task. Similar results were also found by Palmiero and colleagues’ study [51], in which the individual vividness of mental imagery was positively related to the practicality score of the invention in the Mental Synthesis Task. Therefore, the nature of the VCST used in this study and the better predisposition of FIs than FDs in mentally manipulating spatial shapes could represent a possible explanation of our results during the inventive phase. Second, it has been found that the better predisposition of FIs in using their own knowledge and in extracting it from memory, especially in complex tasks in which the solution is unclear, positively affects creative performance [19]. This assumption is consistent with the two-step form of the VCST used in this research. Indeed, unlike the one-step form [29,51] in which the category of creative inventions is specified before combining the visual components, the category is specified in the two-step form only after the assembly of components. This makes the creative process more complex because, at least, in the combination phase, the goal of creative production is not defined, and participants have to adapt what they have previously assembled to the category provided by the task. In other words, during the two-step form of VCST, participants have to reorganize their prior knowledge in order to generate the creative product. Given that FIs, compared with FDs, have a better capacity to extract their own knowledge, this individual predisposition could be helpful to them in reorganizing and updating the structure previously generated in order to generate the creative invention.

Taken together these results showed that field independence positively affected both phases (pre-inventive and inventive) of the creative process. Although one would be expected that FDI differently impacted on the two phases, it is important to acknowledge that, on the one hand, the preinventive phase relies on field independence based on divergent thinking [31,32]; and, on the other hand, the inventive phase relies on field independence based on convergent analytic thinking [36,37]. That is, FIs can encompass the creative process given their ability in shifting between generative/divergent and explorative/convergent phases. This means that real-world creative production based on the Geneplore Model might be fully supported by the field independent cognitive style, given that the latter loads on both the preinventive and inventive phases. Although this interpretation is intriguing, it should be supported by further scientific evidence.

Notably, the VCST—Description length correlated positively to the originality and appropriateness of the inventive scores, meaning that higher the number of words used to describe the objects the higher the originality and appropriateness. This result suggested that the description length may represent a characteristic of creativity [61], especially of the inventive phase of the creative process. Although in this study we did not used a written-narrative task to measure creativity, this result confirmed that when it is necessary to make causal inferences and category reductions, the number of words, reflecting the complexity of the construct [62], can play a key role. Of course, this idea needs also to be verified by future studies.

## 5. Conclusions

To conclude, this research provides empirical evidence on a problematic and complex relationship involving FDI and creativity, and results seem to support the hypothesis that FIs outperform FDs in creative performance. Despite these findings, the current research shows a critical limit concerning the small sample size. This suggests that studies with more subjects should be carried out in order to reach more reliable conclusions. In addition, the field dependent and field independent groups are unbalanced in terms of gender. However, the study provides interesting future directions. First, although the Geneplore model represents a domain-general framework [11], the research focused only on visual real-world creative production. Further studies should encompass different domains of creativity (e.g., verbal and motor domains) in order to define an exhaustive picture of the impact of FDI on creativity. Yet, even though the VCST is not a time-based creativity task, future studies could assess the times necessary to carry on the creative process and related it to the attributes of creativity, such as originality and appropriateness. Then, in the present research, we stressed the pivotal role of mental imagery during the generation and evaluation of preinventive structure as well as during the invention phase. Future investigations could further explore the relationships amongst FDI, creativity, and mental imagery, considering how the vividness of mental images may spur or inhibit this cognitive style’s influence on creative performance.

## Figures and Tables

**Table 1:** Descriptive statistics divided by FDI groups.

	FDs	FIs
N	30	30
Gender	21 F	10 F
Age mean (SD)	21.61 (3.10)	22.41 (3.56)
VCST—Pre—Originality mean (SD)	1.86 (0.37)	2.18 (0.41)
VCST—Pre—Synthesis mean (SD)	1.76 (0.49)	2.25 (0.56)
VCST—Inv—Originality mean (SD)	2.14 (0.59)	2.79 (0.76)
VCST—Inv—Appropriateness mean (SD)	1.97 (0.66)	2.67 (0.81)
VCST—Description length	44.33 (25.66)	59.10 (28.43)

Note. FDs = Field Dependents; FIs = Field Independents; VCST = Visual Creative Synthesis Task; Pre = Preinventive phase; Inv = Inventive phase.

**Table 2:** Means, standard deviations, and Spearman’s Rho inter-correlations.

	*M*	*SD*	1.	2.	3.	4.	5.	6.	7.
1. Gender	-	-	1						
2. EFT (RTs)	41.63	29.25	−0.44 **	1					
3. VCST—Pre—Originality	2.02	0.42	0.34 **	−0.50 **	1				
4. VCST—Pre—Synthesis	2.01	0.57	0.35 **	−0.48 **	0.91 **	1			
5. VCST—Inv—Originality	2.47	0.74	0.27 *	−0.51 **	0.86 **	0.84 **	1		
6. VCST—Inv—Appropriateness	2.34	0.81	0.29 *	−0.52 **	0.82 **	0.85 **	0.93 **	1	
7. VCST—Description length	52.02	27.79	−0.02	−0.30 *	0.15	0.15	0.31 *	0.29 *	1

*Note. N* = 60, gender was dummy coded (F = 0, M = 1) * *p* < 0.05 (two tailed) ** *p* < 0.01 (two tailed) EFT = Embedded Figure Test RTs = Response Times: VCST = Visual Creative Synthesis Task Pre = Preinventive phase Inv = Inventive phase.

**Table 3:** Spearman’s Rho partial correlations.

	1.	2.	3.	4.	5.	6.
1. EFT (RTs)	1					
2. VCST—Pre—Originality	−0.41 **	1				
3. VCST—Pre—Synthesis	−0.38 **	0.90 **	1			
4. VCST—Inv—Originality	−0.46 **	0.85 **	0.83 **	1		
5. VCST—Inv—Appropriateness	−0.46 **	0.80 **	0.84 **	0.93 **	1	
6. VCST—Description length	−0.35 **	0.16	0.17	0.33 *	0.31 *	1

*Note. N* = 60, * *p* < 0.05 (two tailed) ** *p* < 0.01 (two tailed) EFT = Embedded Figure Test RTs = Response Times: VCST = Visual Creative Synthesis Task Pre = Preinventive phase Inv = Inventive phase.

## Data Availability

The data presented in this study are available on request from the corresponding author. The data are not publicly available due to subject confidentiality.

## References

[1] Cropley A.J. (1990). Creativity and mental health in everyday life. Creat. Res. J..

[2] Kaufman J.C., Beghetto R.A. (2009). Beyond big and little: The four c model of creativity. Rev. Gen. Psychol..

[3] Guilford J.P. (1967). Creativity: Yesterday, today and tomorrow. J. Creat. Behav..

[4] Runco M.A., Acar S. (2012). Divergent thinking as an indicator of creative potential. Creat. Res. J..

[5] Amabile T.M. (1988). A model of creativity and innovation in organizations. Res. Organ. Behav..

[6] Sternberg R.J., Sternberg R.J. (1988). A three-facet model of creativity. The Nature of Creativity: Contemporary Psychological Perspectives.

[7] Finke R.A., Ward T.B., Smith S.M. (1992). Creative Cognition: Theory, Research, and Applications.

[8] Gruszka A., Tang M., Tang M., Werner C.H. (2017). The 4P’s creativity model and its application in different fields. Handbook of the Management of Creativity and Innovation: Theory and Practice.

[9] Ward T.B. (2001). Creative cognition, conceptual combination, and the creative writing of Stephen R. Donaldson. Am. Psychol..

[10] Finke R.A. (1996). Imagery, creativity, and emergent structure. Conscious. Cogn..

[11] Kleinmintz O.M., Ivancovsky T., Shamay-Tsoory S.G. (2019). The two-fold model of creativity: The neural underpinnings of the generation and evaluation of creative ideas. Curr. Opin. Behav. Sci..

[12] Kaufman J.C., Sternberg R.J. (2010). The Cambridge Handbook of Creativity.

[13] Sternberg R.J., Lubart T.I. (1991). An investment theory of creativity and its development. Hum. Dev..

[14] Abraham A. (2018). The Neuroscience of Creativity.

[15] Runco M.A., Jaeger G.J. (2012). The standard definition of creativity. Creat. Res. J..

[16] Ward T.B., Kennedy E.S. (2017). Creativity research: More studies, greater sophistication and the importance of “big” questions. J. Creat. Behav..

[17] Lei W., Deng W., Zhu R., Runco M.A., Dai D.Y., Hu W. (2020). Does Cognitive Style Moderate Expected Evaluation and Adolescents’ Creative Performance: An Empirical Study. J. Creat. Behav..

[18] Riding R., Rayner S. (2013). Cognitive Styles and Learning Strategies: Understanding Style Differences in Learning and Behavior.

[19] Li C., Mu X., Tan Y., Gu C., Hu B.Y., Fan C. (2020). Do field-dependent individuals tend to have lower creativity than field-independent ones? The role of informational cues in electronic brainstorming. Interact. Learn. Environ..

[20] Witkin H.A., Moore C.A., Goodenough D.R., Cox P.W. (1977). Field-dependent and field-independent cognitive styles and their educational implications. Rev. Educ. Res..

[21] Zhang L.F. (2017). The Value of Intellectual Styles.

[22] Zeng X.Q., Liu J.P., Chen M.R. (2010). The relationship between cognitive style and implicit/explicit memory. J. Psychol. Sci..

[23] Zhang L.F. (2004). Field-dependence/independence: Cognitive style or perceptual ability? Validating against thinking styles and academic achievement. Pers. Individ. Differ..

[24] Guisande M.A., Páramo M.F., Tinajero C., Almeida L.S. (2007). Field dependence-independence (FDI) cognitive style: An analysis of attentional functioning. Psicothema.

[25] Gerwig A., Miroshnik K., Forthmann B., Benedek M., Karwowski M., Holling H. (2021). The relationship between intelligence and divergent thinking—A meta-analytic update. J. Intell..

[26] Palmiero M., Fusi G., Crepaldi M., Borsa V.M., Rusconi M.L. (2022). Divergent thinking and the core executive functions: A state-of-the-art review. Cogn. Process..

[27] Lee L.Y., Talhelm T., Zhang X., Hu B., Lv X. (2021). Holistic thinkers process divided-attention tasks faster: From the global/local perspective. Curr. Psychol..

[28] Reiter-Palmon R., Forthmann B., Barbot B. (2019). Scoring divergent thinking tests: A review and systematic framework. Psychol. Aesthet. Creat. Arts.

[29] Giancola M., Palmiero M., D’Amico S. (2022). Exploring the interplay between fluid intelligence and creativity: The mediating role of the field-dependent-independent cognitive style. Think. Ski. Creat..

[30] Giancola M., Palmiero M., D’Amico S. (2022). Field dependent-independent cognitive style and creativity from the process and product-oriented approaches: A systematic review. Creat. Stud..

[31] Spotts J.V., Mackler B. (1967). Relationships of field-dependent and field-independent cognitive styles to creative test performance. Percept. Mot. Ski..

[32] Nisiforou E.A. Examining the association between users creative thinking and field dependence-independence cognitive style through eye movement components. Proceedings of the ACM SIGCHI Conference on Creativity and Cognition.

[33] Bloomberg M. (1971). Creativity as related to field independence and mobility. J. Genet. Psychol..

[34] Niaz M., De Nunez G.S., De Pineda I.R. (2000). Academic performance of high school students as a function of mental capacity, cognitive style, mobility-fixity dimension, and creativity. J. Creat. Behav..

[35] Bal S. (1988). Creativity, cognitive style and academic achievement amongst university students. Psychol. Stud..

[36] Noppe L.D., Gallagher J.M. (1977). A cognitive style approach to creative thought. J. Pers. Assess..

[37] Chadha N.K. (1985). Creativity and cognitive style. Psycho-Lingua.

[38] Ohnmacht F.W., McMorris R.F. (1971). Creativity as a function of field independence and dogmatism. J. Psychol..

[39] Miller A.L. (2007). Creativity and cognitive style: The relationship between field-dependence-independence, expected evaluation, and creative performance. Psychol. Aesthet. Creat. Arts.

[40] Fergusson L.C. (1992). Field independence and art achievement in meditating and nonmeditating college students. Percept. Mot. Ski..

[41] Fergusson L.C. (1993). Field independence, transcendental meditation, and achievement in college art: A reexamination. Percept. Mot. Ski..

[42] Jaarsveld S., Lachmann T. (2017). Intelligence and creativity in problem solving: The importance of test features in cognition research. Front. Psychol..

[43] Fogliani T., Di Nuovo S., Fogliani A.M., Pizzamiglio L. (1984). Dipendenza Dal Campo e Stile Cognitivo: Gli Embedded Figures Tests Di H. Witkin, PK Oltman, E. Raskin e SA Karp.

[44] Palmiero M., Nori R., Aloisi V., Ferrara M., Piccardi L. (2015). Domain-specificity of creativity: A study on the relationship between visual creativity and visual mental imagery. Front. Psychol..

[45] Amabile T.M. (1982). Social psychology of creativity: A consensual assessment technique. J. Pers. Soc. Psychol..

[46] Tascón L., Boccia M., Piccardi L., Cimadevilla J.M. (2017). Differences in spatial memory recognition due to cognitive style. Front. Pharmacol..

[47] Niaz M., De Nunez G.S. (1991). The relationship of mobility-fixity to creativity formal reasoning and intelligence. J. Creat. Behav..

[48] Teghil A., Boccia M., Guariglia C. (2019). Field dependence–independence differently affects retrospective time estimation and flicker-induced time dilation. Exp. Brain Res..

[49] Mefoh P.C., Nwoke M.B., Chukwuorji J.C., Chijioke A.O. (2017). Effect of cognitive style and gender on adolescents’ problem solving ability. Think. Ski. Creat..

[50] Palmiero M., Nakatani C., Raver D., Belardinelli M.O., van Leeuwen C. (2010). Abilities within and across visual and verbal domains: How specific is their influence on creativity?. Creat. Res. J..

[51] Palmiero M., Cardi V., Belardinelli M.O. (2011). The role of vividness of visual mental imagery on different dimensions of creativity. Creat. Res. J..

[52] Palmiero M., Nori R., Piccardi L. (2016). Visualizer cognitive style enhances visual creativity. Neurosci. Lett..

[53] Sack A.T., Jacobs C., De Martino F., Staeren N., Goebel R., Formisano E. (2008). Dynamic premotor-to-parietal interactions during spatial imagery. J. Neurosci..

[54] Roskos-ewoldsen B., Black S.R., McCown S.M. (2008). Age-related changes in creative thinking. J. Creat. Behav..

[55] Finke R.A., Pinker S., Farah M.J. (1989). Reinterpreting visual patterns in mental imagery. Cogn. Sci..

[56] Palmiero M., Piccardi L. (2020). Is Visual Creativity Embodied? Thinking Aloud while Performing the Creative Mental Synthesis Task. Brain Sci..

[57] Hindal H., Reid N., Badgaish M. (2009). Working memory, performance and learner characteristics. Res. Sci. Technol..

[58] Evans C., Richardson J.T., Waring M. (2013). Field independence: Reviewing the evidence. Br. J. Educ. Psychol..

[59] Boccia M., Piccardi L., Di Marco M., Pizzamiglio L., Guariglia C. (2016). Does field independence predict visuo-spatial abilities underpinning human navigation? Behavioural evidence. Exp. Brain Res..

[60] Li H., Zhang Y., Wu C., Mei D. (2016). Effects of field dependence-independence and frame of reference on navigation performance using multi-dimensional elec-tronic maps. Pers. Individ. Differ..

[61] Taylor C.L., Kaufman J.C., Barbot B. (2021). Measuring creative writing with the storyboard task: The role of effort and story length. J. Creat. Behav..

[62] Burleson B.R., Samter W., Waltman M.S. (1987). More evidence that cognitive complexity is not loquacity: A reply to beatty and payne. Commun. Q..

